# Non-structural carbohydrate profiles and ratios between soluble sugars and starch serve as indicators of productivity for a bioenergy grass

**DOI:** 10.1093/aobpla/plv032

**Published:** 2015-05-12

**Authors:** Sarah Jane Purdy, Anne Louise Maddison, Jennifer Cunniff, Iain Donnison, John Clifton-Brown

**Affiliations:** 1 Institute of Biological Environmental and Rural Sciences, Aberystwyth University, Plas Gogerddan, Ceredigion SY23 3EE, UK; 2 Rothamsted Research, Harpenden, Hertfordshire AL5 2JQ, UK

**Keywords:** Bioenergy, biomarkers, carbohydrate partitioning, carbohydrates, metabolism, *Miscanthus*

## Abstract

There is a pressing need to find a sustainable alternative to fossil fuels that will not compromise food security or require extensive use of agrochemicals. *Miscanthus* is a perennial energy grass predominantly used for combustion but with the current advancement of ligno-cellulosic fermentation technologies there is an interest in using *Miscanthus* for bioethanol production. Currently, the only commercially grown genotype of *Miscanthus* is *M.*× *giganteus*; a high yielding, interspecific hybrid of *M. sacchariflorus* and *M. sinensis*. As *M.*× *giganteus* is a sterile triploid, it cannot be used as a parent so *Miscanthus* breeding effort is focused on producing new interspecific varieties that out-perform *M.*× *giganteus*. The carbohydrate profiles of four genotypes of *Miscanthus*, including *M. sacchariflorus* (Sac-5), *M.*× *giganteus* (Gig-311), *M. sinensis* (Sin-11) and *M. sinensis* (Goliath), were characterized at replicated field sites in Aberystwyth, West Wales and Harpenden, south-east England. Our hypothesis was that a distinctive carbohydrate profile underlies enhanced biomass accumulation. Biomass accumulation is greatest when day-lengths and solar intensity are highest; so, observations were made in the middle of UK summer (July) for 2 years. Gig-311 had a greater abundance of fructose in its stems at both sites, and both Gig-311 and Sac-5 had low abundance of starch. At both sites, the highest yielding genotype was Gig-311 and Sac-5 was also high yielding at Harpenden, but performed comparatively poorly at Aberystwyth. At both sites Gig-311 had a distinctly high concentration of fructose, low starch and a high ratio of soluble sugars: starch, and at Harpenden, Sac-5 was similar. We conclude that the abundance of starch and fructose and a greater partitioning of soluble sugars, relative to starch, are candidate biomarkers of productivity in *Miscanthus*.

## Abstract (precis)

Miscanthus is a perennial bioenergy crop that offers a sustainable alternative to fossil fuels. We sought to identify candidate metabolic biomarkers of productivity that may be used as a method of screening for superior individuals in breeding programmes. Our experiments were carried out over two years and two sites in four genotypes. The concentration of fructose positively correlated whereas starch and the ratio of soluble sugars to starch negatively correlated with three biomass traits: yield, stem height and growth rate. Our results show the potential of the carbohydrate metabolic profile as a biomarker of productivity in a perennial energy crop.

## Introduction

There is a global urgency to replace fossil fuels with a sustainable source of bioenergy without compromising soil health, food security or requiring the heavy use of agrochemicals. Dedicated energy crops can produce high yields with low inputs and therefore provide a means by which sustainable energy can be generated. The use of perennial crops can also improve water retention, increase climate change adaptation and promote biodiversity compared with traditional annual systems (Wang and Alonzo 2013).

*Miscanthus* is a giant perennial grass native to Eastern Asia but cultivated for bioenergy production in Europe and North America. Currently, *Miscanthus* is mainly used for combustion as a substitute for coal, but with the advancement of ligno-cellulosic fermentation technologies there is an increasing interest in using *Miscanthus* as a sustainable source of bioethanol (Visser and Pignatelli 2001; Hodgson *et al*. 2010; Somerville *et al*. 2010). Despite a large range of diversity in *Miscanthus*, currently only a single genotype is grown in commercial plantations in Europe and the USA, *M.*× *giganteus. Miscanthus*× *giganteus* is a triploid interspecific hybrid, derived from a cross between a tetraploid *M. sacchariflorus* and a diploid *M. sinensis*. As *M.*× *giganteus* is a sterile triploid, no improvements to the yield potential of *Miscanthus* can be achieved through using this genotype as a parent plant. Therefore, current breeding efforts are focussed on producing new varieties that can out-perform *M.*× *giganteus*. As an exceptional genotype derived from an interspecific cross, there is particular interest in re-creating similar crossing events to release hybrid vigour in the resulting progeny. Certainly, *Miscanthus* exhibits strong heterosis; in a study of 244 genotypes containing *M. sinensis*, *M. sacchariflorus* and hybrids, the highest yielding genotypes were the triploids and interspecific hybrids (Robson *et al*. 2013).

*Miscanthus* takes 3 years to reach maturity and it is an outcrossing species. These factors greatly extend the time required to produce new varieties compared with inbreeding annual species. In order to increase the speed at which new varieties can be produced, marker-assisted selection (MAS) is being developed for *Miscanthus* (Slavov *et al*. 2013). In addition to molecular markers, the metabolome has been demonstrated to act as a marker of plant productivity in three separate studies on *Arabidopsis thaliana* (*Arabidopsis*). In the first study, combinations of metabolites were found to correlate with biomass; metabolites of particular significance were intermediates of the hexose pool such as fructose-6-phosphate, members of the tricarboxylic acid cycle and sucrose (Meyer *et al*. 2007). All of these metabolites negatively correlated with biomass, suggesting that they were reduced to low concentrations during periods of rapid growth (Meyer *et al*. 2007). In the second study, negative correlations between biomass, leaf starch abundance and protein content were detected and from these observations the authors used a transcriptomics approach to identify two genes whose transcripts correlated with biomass (Sulpice *et al*. 2009). By correlating a negative relationship with starch and a positive correlation with enzyme activity, approximately a third of the variation in biomass of an *Arabidopsis* inbred family could be accounted for in a third study (Sulpice *et al*. 2010). The relationships between metabolites can also be indicative of plant performance, for example changes in the ratio of chlorophyll *a* : *b* occur during cold stress and an increase in the glycine : serine ratio indicates an impairment of the oxidative photosynthetic carbon cycle (Diaz *et al*. 2005; Purdy *et al*. 2013*a*). There is also evidence that the ratio of sucrose to starch may be indicative of biomass potential. The immediate products of photosynthesis are partitioned between sucrose, which can be immediately metabolized to fuel growth, and starch which provides transient storage for metabolism overnight (Smith and Stitt 2007). In more rapidly growing accessions of *Arabidopsis*, less starch was retained at the end of the night, suggesting that a slightly larger proportion of carbohydrate had been partitioned into sucrose rather than starch (Cross *et al*. 2006; Smith and Stitt 2007). These studies show that metabolites and relationships or ratios between them can be used as biomarkers of biomass potential and/or indicate stress responses in the model plant, *Arabidopsis*.

We aimed to identify potential markers of productivity in *Miscanthus*. As several previous reports in other species had successfully used non-structural carbohydrates as indicators of performance, we also focussed on these metabolites. The questions we asked were:

1. Does the abundance of a particular carbohydrate, such as starch, or a suite of carbohydrates correlate with biomass traits?
2. Does the partitioning of carbohydrate between different pools, e.g. starch and sucrose correlate with biomass traits in *Miscanthus* as has been reported in *Arabidopsis*?
3. Does the partitioning of carbohydrates between the leaf and stem differ in high or low yielding genotypes?

Metabolic biomarkers are routinely used in medicine, but they have rarely been used as a predictive tool in plant science, particularly in non-model species. In perennial species that take years to reach maturity and for which molecular technologies do not yet exist, metabolic biomarkers could be a valuable breeding tool with which to screen new crosses.

Our experiment utilized four clones of *Miscanthus*, including the highly productive genotype *M.*× *giganteus*, sampled around mid-summer over 2 years. Mid-summer was chosen as the harvest time point because this corresponds to the longest day-lengths and highest solar intensity which promotes rapid growth (biomass accumulation).

## Methods

### Ethics statement

The field trials described in this paper were established on private land belonging to the Institute of Biological Environmental and Rural Sciences (IBERS), Aberystwyth, West Wales (52.4139′N, −4.014′W) and Rothamsted Research in Harpenden, south-east England (51.82′N, 0.38′W). Both sites are on land dedicated for agricultural research so no specific permission was required to establish the trials. No endangered or protected species were affected either by the establishment of the trials or subsequent experimental work.

### Plant material

**Figure 1.**
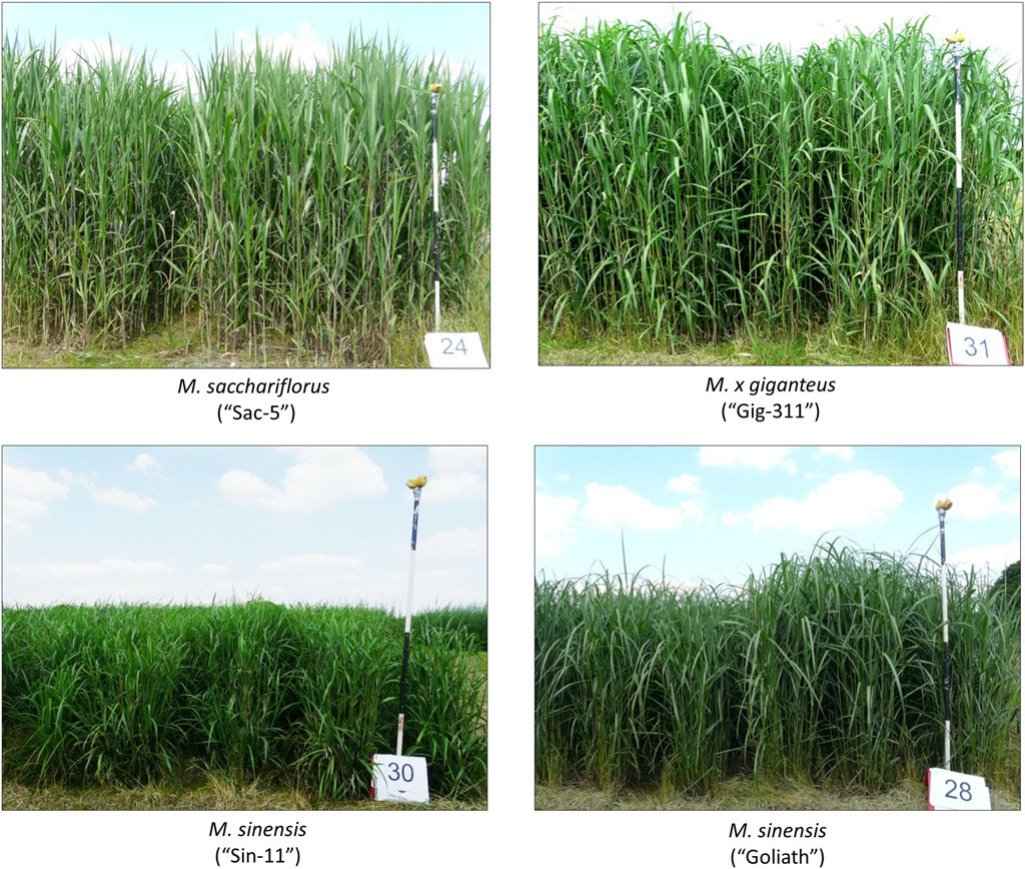
The physical appearance of the four genotypes used in this study. The measuring stick in the right of each image is 2 m tall. Plants were photographed at Harpenden in July 2012.

All genotypes used in this study are of Japanese origin. *Miscanthus sinensis* (Sin-11) is a diploid clone selected in 1988 from temperate Japan (Honshu Island) by Danish plant collector Dr Poul Erik Brander. It was part of the European *Miscanthus* Improvement (EMI) programme (1997–2000) and is the female parent of the Mx2 mapping family (Ma *et al*. 2012). This genotype was previously called ‘EMI-11’ (Purdy *et al*. 2013*b*). *Miscanthus sinensis* (Goliath) is a triploid intraspecific hybrid of *M. sinensis* and was originally selected as a vigourous seedling from a cross (parents unknown) by Ernst Pagels and marketed as a ‘large-type’ horticultural variety since the 1970s. *Miscanthus sacchariflorus* (Sac-5) is a tetraploid that was part of a seed population collected from central Japan, by TINPLANT, in 1992. It was also part of the EMI programme. *Miscanthus*× *giganteus* (Gig-311) is a naturally occurring triploid hybrid of diploid *M. sinensis* and a tetraploid *M. sacchariflorus*. It was supplied to Aberystwyth from Bical Farms Ltd (Taunton) in 2005. The physical appearance of the four genotypes in July can be seen in Fig. 1.

### Establishment of the field trials

Two dedicated trials were established in May 2009 as part of the BSBEC-BioMASS project (http://www.bsbec-biomass.org.uk/) at the Institute of Biological Environmental and Rural Sciences (IBERS) West Wales (52.4139′N, −4.014′W) and Rothamsted Research in Harpenden, south-east England (51.82′N, 0.38′W). At both sites plants were arranged in randomized block designs consisting of four blocks, each block containing four plots, one for each *Miscanthus* genotype described above. Each plot contained 121 plants (7.8× 7.8 m^2^) with areas designated for: non-destructive measurements, annual yield harvest and destructive harvests. Plants were grown from rhizome pieces cut from mature stands in modules before planting at a density of 2 plants m^−2^. Surrounding each plot was a row of guard plants of the same genotype. The soil type at Aberystwyth is classified as a silty clay loam. The soil type at Harpenden is classified as a silty clay loam (18–27 % clay) with high flint content. Prior to the establishment of the BSBEC field trial, the Aberystwyth site was under grassland and the Harpenden site in cereal cultivation. Both sites were ploughed, power harrowed and then planted. The surrounding paths were then re-sown with grass.

### Climatic measurements

Meteorological measurements were monitored by on-site weather stations (Campbell Scientific Ltd, Shepshed, UK) fitted with a CR1000 data-logger and a multiplexer. Rainfall was recorded on-site at Aberystwyth whereas at Harpenden these data were obtained from the central weather station located 1.7 km from the site across flat ground.

### In season destructive harvests

As follows, plants were harvested in July 2011 and July 2012: plants within the designated destructive harvest area in each plot were assigned a number and a single plant selected using a random number generator. At each destructive time point, a single plant per plot was harvested (*n* = 4). It is recognized in a number of species, including *Miscanthus*, that strong diurnal flux in carbohydrate occurs (Sicher *et al*. 1984; Hendrix and Huber 1986; Purdy *et al*. 2013*b*). Owing to these changes destructive harvests had to be completed within a minimum timeframe. Therefore, to avoid confounding diurnal effects, the sites were harvested over 2 days; on Day 1, blocks 1 and 3 were harvested and on Day 2, blocks 2 and 4 were harvested. This enabled all harvests to be completed within a 2 h window either side of the solar noon. For each destructively harvested plant, the tallest stem was harvested at 10 cm from the soil surface. The leaves were removed and the fresh weight of both stem and leaves were recorded separately before flash freezing in liquid nitrogen and storing on dry ice. The remaining total aboveground biomass was then harvested at 10 cm, the material was chipped and a sub-sample taken, flash frozen and stored on dry ice. Therefore, a total of three samples were taken from each plant: leaf, stem and total aboveground biomass. The tallest stem and aboveground sub-sample were freeze dried to a constant weight, and the tallest stem was then weighed to determine the biomass (dry weight). Plant tissues were course milled and then cryomilled for subsequent analysis.

### End-of-season yield harvests

*Miscanthus* is normally harvested for bioenergy in winter or early spring when the crop has senesced and is at its lowest moisture content. Harvests were carried out in January 2012 and January 2013, following the 2011 and 2012 growing seasons, respectively. The yield area of each plot comprised of 12 plants which were harvested by hand, pooled then weighed, sub-sampled and oven dried to determine the total dry weight yield.

### Phenotypic measurements

Throughout the months of June and July, measurements of canopy height were made fortnightly. The average growth rate for the 2-weeks preceding the destructive harvest was then calculated as the change in height divided by the number of days (cm day^−1^). Measurements were made on the same four plants per plot over the 8-week period. These values were then averaged on a per plot basis to provide a single value per plot to avoid pseudoreplication.

### Non-structural carbohydrate compositional analyses

Carbohydrate compositional analysis was carried out as previously described (Purdy *et al*. 2013*b*). Soluble sugar extraction: ∼20 mg (actual weight recorded) of each cryomilled (6870 Freezer Mill, Spex, Sampleprep, Stanmore, UK) plant tissue sample was weighed into 2 mL screw cap micro-centrifuge tubes. Sugars were extracted four times with 1 mL of 80 % (v/v) ethanol and the resulting supernatants pooled; two extractions were at 80 °C for 20 and 10 min, respectively, and the remaining two at room temperature. A 0.5 mL aliquot of soluble sugar extract and the remaining pellet containing the insoluble fraction (including starch) were dried down in a centrifugal evaporator (Jouan RC 1022, Saint Nazaire, France) until all the solvent had evaporated. The dried down residue from the soluble fraction was then re-suspended in 0.5 mL of distilled water. Samples were stored at −20 °C for analysis.

#### Soluble sugar analysis

Soluble sugars of samples extracted in the previous step were quantified enzymatically by the stepwise addition of hexokinase, phosphoglucose isomerase and invertase (INV) (Jones *et al*. 1977). Samples were quantified photometrically (Ultraspec 4000, Pharmacia Biotech, Sweden) by measuring the change in wavelength at 340 nm for 20 min after the addition of each enzyme. Sucrose, glucose and fructose were then quantified from standard curves included on each 96-well plate.

#### Starch quantification

Starch was quantified using a modified Megazyme protocol (Megazyme Total Starch Assay Procedure, AOAC method 996.11, Megazyme International, Ireland). Briefly, the dried pellet was resuspended in 0.4 mL of 0.2 M KOH, vortexed vigorously and heated to 90 °C in a water bath for 15 min to facilitate gelatinization of the starch. A total of 1.28 mL of 0.15 M NaOAc (pH 3.8) was added to each tube (to neutralize the sample) before the addition of 20 µL α-amylase and 20 µL amyloglucosidase (Megazyme International, Ireland). After incubation at 50 °C for 30 min and centrifugation for 5 min, a 0.02 mL aliquot was combined with 0.6 mL of GOPOD reagent (Megazyme). A total of 0.2 mL of this reaction was assayed photometrically (Ultraspec 4000, Pharmacia Biotech, Sweden) on a 96-well microplate at 510 nm against a water-only blank. Starch was quantified from known standard curves on the same plate. Each sample and standard was tested in duplicate. Each plate contained a *Miscanthus* control sample of known concentration for both soluble sugars and starch analysis.

### Statistical analysis

Statistical analyses were carried out using GenStat Version 13 (VSN International Ltd, Rothamsted Research, Harpenden and Numerical Algorithmns Group, Oxford, UK). To identify genotypic and annual differences in biomass traits and total aboveground carbohydrates, two-way analyses of variance (ANOVA) were used for each site in which year (2011 and 2012) and the four genotypes were grouped as factors. Associated Tukey honestly significant difference (HSD) tests were performed to identify specific differences between genotypes for each year's data (*n* = 4). To identify differences in leaf and stem carbohydrate concentrations and ratios, data for Aberystwyth from both years was used in a single analysis (2 years× 4 reps, *n* = 8) and the value of the eight individual plants harvested per genotype was used to generate the mean and SE. At Harpenden, only a single year was analysed for leaf and stem carbohydrate concentration (2012) and therefore *n* = 3–4. For all statistical tests, significant differences equal *P*≤0.05. Regression analyses were performed in Excel, significant correlations were determined by ANOVA, *P*≤0.05.

## Results

### Biomass traits

The physical appearances of the four genotypes used in this study are shown in Fig. 1. In 2011 Gig-311 was the fastest growing genotype at Aberystwyth growing >3 cm day^−1^, no differences between genotypes were observed at Harpenden (Table 1). In 2012, Sac-5 showed a similar rate of growth to Gig-311 and both these genotypes showed a more rapid rate of growth than Sin-11 and Goliath at both sites (Table 1). The genotype with the greatest canopy height in 2011 was Gig-311 at Aberystwyth, whereas no significant differences were observed at Harpenden. At Aberystwyth, in 2012, the canopy height of Sac-5 was greater than the two *M. sinensis* genotypes which was similar to Harpenden where Sac-5 was significantly taller than Sin-11. At the annual yield harvest in January 2012 and 2013, Gig-311 attained significantly more biomass than the other genotypes in both years at Aberystwyth but only in 2013 at Harpenden. Sac-5 also yielded well at Harpenden in 2013, producing higher yields than all genotypes at Aberystwyth except Gig-311 (Table 1). The lack of significant differences between genotypes at Harpenden in 2011 can be explained by the climatic data from that year, where Harpenden was subject to a drought event in spring 2011 **[see Supporting Information—Fig. S1]**. Rainfall in Spring at Harpenden was <20 mm in May and although it recovered in June, rainfall declined to below average again in July. Aberystwyth rainfall was equal to, or greater than, the 19-year average during May–July 2011 [**see Supporting Information****—Fig. S1**]. In 2012 rainfall was greater than the 19-year average at both sites. We therefore suggest that in July 2011 the plants had been affected by drought in Harpenden which reduced their growth rates and yield. For this reason the subsequent carbohydrate analysis is presented only for plants grown at Harpenden in 2012.

**Table 1.** The average growth rate (cm day^−1^) and canopy height (cm) of the plants destructively harvested in July 2011 and 2012 and the final yield harvests at the end of each respective growing season in January 2012 and January 2013 (g DW plant^−1^) at Aberystwyth (A) and Harpenden (B). *N* = 3–4 ± SE. Different letters show significant differences (Tukey's HSD test *P* ≤ 0.05) and statistical analysis is a two-way ANOVA (*P* ≤ 0.05).

	Growth rate		Canopy height		Final biomass	
	2011	2012	2011	2012	2011	2012
**(A) Aberystwyth**						
Sac-5	1.6 ± 0.1a	3.2 ± 0.2a	142.0 ± 8.7a	208.9 ± 19.1a	258.3 ± 41.6a	478.2 ± 21.4a
Gig-311	3.5 ± 0.2b	3.5 ± 0.2a	225.1 ± 8.7b	177.0 ± 26.8ab	803.9 ± 139.5b	729.2 ± 46.5b
Sin-11	1.5 ± 0.2a	1.5 ± 0.2b	101.3 ± 5.7c	115.6 ± 3.4b	237.4 ± 55.1a	403.3 ± 44.5a
Goliath	1.4 ± 0.2a	1.9 ± 0.1b	132.6 ± 6.6ac	136.4 ± 10.4b	442.4 ± 25.0a	546.4 ± 29.4a
Genotype	<0.001		<0.001		<0.001	
Year	<0.001		0.341		0.025	
Geno × Year	<0.001		0.001		0.039	
**(B) Harpenden**						
Sac-5	0.6 ± 0.2a	3.9 ± 0.4a	196.8 ± 24.6a	170.8 ± 19.7a	390.8 ± 101.9a	673.4 ± 89.7ab
Gig-311	1.1 ± 0.1a	3.5 ± 0.3a	129.3 ± 38.7a	171.3 ± 19.5ab	439.5 ± 96a	897.4 ± 45.8b
Sin-11	1.2 ± 0.3a	2 ± 0.2b	128.6 ± 12.9a	112.4 ± 11.4b	305.4 ± 57.2a	452.4 ± 38.2a
Goliath	0.6 ± 0.3a	1.9 ± 0.2b	121.3 ± 6.5a	95.3 ± 4.1ab	436.2 ± 77.2a	567.8 ± 48.3a
Genotype	0.003		<0.001		0.012	
Year	<0.001		<0.001		<0.001	
Geno × Year	<0.001		0.003		0.174	

### Carbohydrate composition

**Figure 2.**
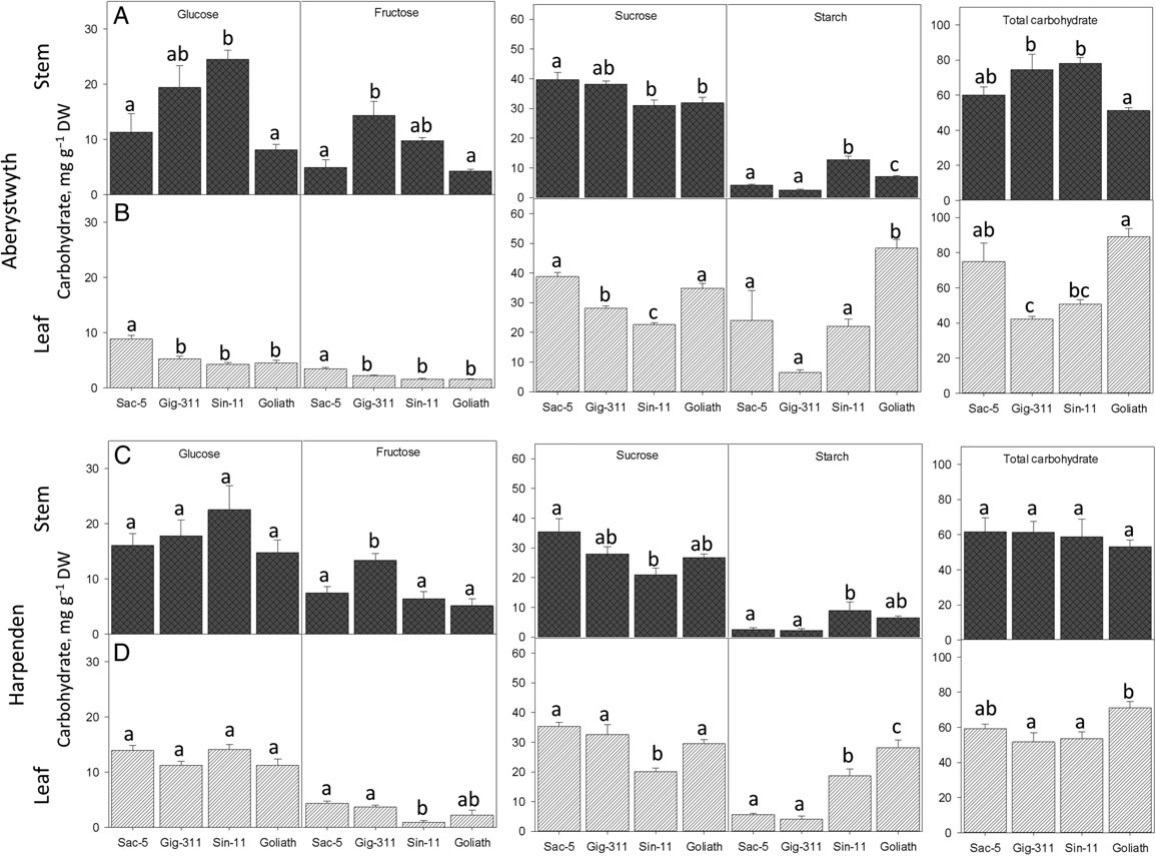
(A–D) The partitioning of carbohydrate (mg g^−1^ DW) in between the stem (A and C) and the leaf (B and D) at Aberystwyth (A and B) and Harpenden (C and D). Data for Aberystwyth are the average of 2011 and 2012 levels and Harpenden data are from 2012. *N* = 8 (Aberystwyth) and *N* = 3–4 (Harpenden) ±SE. Different letters above the bars show significant differences (Tukey's HSD test *P* ≤ 0.05).

**Figure 3.**
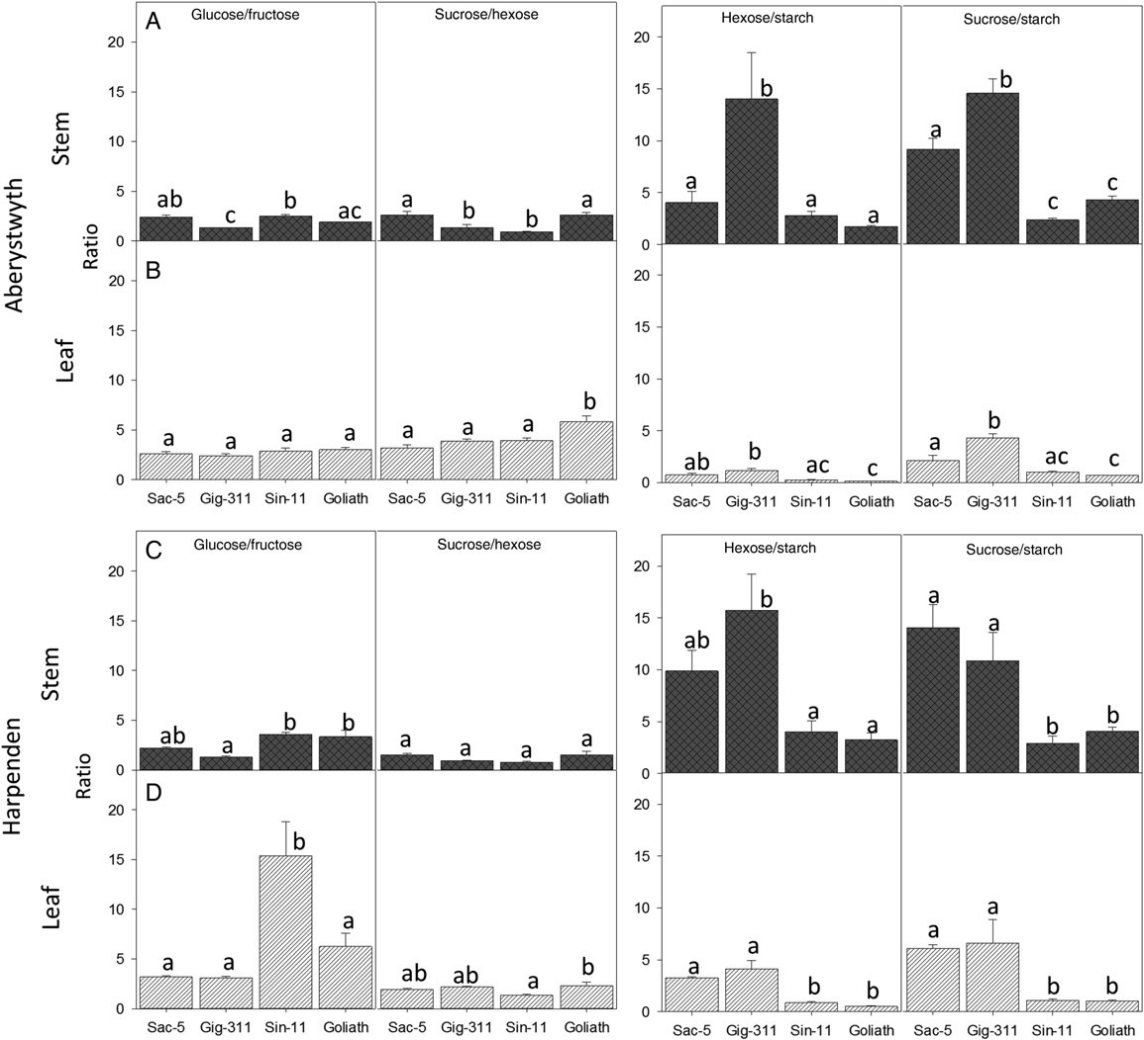
(A–D) The ratio of different pools of carbohydrate partitioned between the stem (A and C) and the leaf (B and D) at Aberystwyth (A and B) and Harpenden (C and D). Data are the average of 2011 and 2012 levels for Aberystwyth and 2012 for Harpenden. *N* = 8 at Aberystwyth and *N* = 3–4 at Harpenden ±SE. Different letters above the bars show significant differences (Tukey's HSD test *P* ≤ 0.05).

The concentration of carbohydrates in the total aboveground biomass was largely consistent between years at Aberystwyth, but not at Harpenden **[see Supporting Information—Table S1]**. As carbohydrate concentrations were similar between years at Aberystwyth, the average of the 2 years was calculated for subsequent analyses (Figs 2 and 3) but only 2012 values were used for Harpenden owing to the drought that affected the site in 2011.

The trends in carbohydrate abundance of the four genotypes were strikingly similar between sites, particularly in the stem tissues (Fig. 2). The four genotypes contained a higher concentration of soluble carbohydrates in their stem tissues than in the leaf at both sites (Fig. 2). At both sites the concentration of fructose in the stems of Gig-311 was significantly greater than that in the stems of Sac-5 and Goliath (Fig. 2A and C) and at both sites the concentration of fructose in the leaf of Sac-5 was greater than that in the leaf of Sin-11. At both sites sucrose concentration was higher in Sac-5 than that in Sin-11 in both leaf and stem. Starch was the only carbohydrate to be in greater abundance in the leaf rather than in the stem. Sin-11 had the greatest abundance of starch in the stem and Goliath had the greatest abundance in the leaf at both sites (Fig. 2). Gig-311 had the lowest concentration of starch in both leaf and stem at both sites but was not significantly different to Sac-5. Slightly more of the total carbohydrate was partitioned in the stem in Gig-311 and Sin-11 at Aberystwyth, whereas Sac-5 and Goliath had a significantly higher concentration of total carbohydrates in the leaf. A similar trend in the leaf was observed at Harpenden but no differences between genotypes were observed in the stem (Fig. 2).

At both sites the ratio of glucose, fructose was lowest and the ratio of hexose : starch was greatest in the stem tissues of Gig-311 (Fig. 3). At Harpenden, Gig-311 and Sac-5 tended to be similar to each other but different from the other genotypes whereas at Aberystwyth Gig-311 was different to all other genotypes (Fig. 3).

Linear regression analyses were carried out for the different carbohydrate concentrations and ratios between them for the three biomass traits at each site (Table 2). Significant correlations (*R*^2^, *P* ≤ 0.05) that were consistent between sites included a negative correlation of −0.4–0.5 between stem starch and both growth rate and final yield, and a positive correlation between leaf fructose and growth rate. The ratios of hexose : starch and sucrose : starch showed significantly positive correlations of 0.3–0.7 for all biomass traits in both tissue types at both sites (with the exception of the hexose : starch in the leaf material and final yield at Aberystwyth) (Table 2). Therefore, the concentration of fructose in the stem and the ratio of hexose : starch and sucrose : starch may be good biomarkers of yield potential.

**Table 2.** Linear regression analysis of carbohydrate concentrations and ratios against the biomass traits in the leaf and stem in 2012. *N* = 15–16. Bold values show significant correlations (ANOVA, *P* ≤ 0.05). Significant negative correlations are pre-fixed with a minus symbol (−).

	Correlation *R*^2^					
	Growth rate		Canopy height		Biomass yield	
	Stem	Leaf	Stem	Leaf	Stem	Leaf
**Aberystwyth**						
Carbohydrate						
Glucose	0.00	**0**.**25**	0.06	**0**.**30**	0.00	0.00
Fructose	0.10	**0**.**27**	0.14	0.12	0.22	0.00
Sucrose	0.12	0.14	0.17	**0**.**35**	0.05	0.00
Starch	**−0**.**53**	0.10	0.18	0.03	**−0**.**50**	0.17
Total carbohydrate	0.00	0.00	0.07	0.01	0.00	0.11
Ratio						
Glucose/fructose	0.15	0.04	0.00	0.08	0.65	0.03
Sucrose/hexose	0.00	0.21	0.00	0.07	0.00	0.00
Hexose/starch	**0**.**39**	**0**.**41**	**0**.**27**	**0**.**24**	**0**.**55**	0.12
Sucrose/starch	**0**.**64**	**0**.**55**	**0**.**24**	**0**.**33**	**0**.**69**	**0**.**42**
**Harpenden**						
Carbohydrate						
Glucose	0.02	0.01	0.07	0.23	0.06	0.02
Fructose	0.17	**0**.**44**	**0**.**34**	0.12	0.18	**0**.**28**
Sucrose	0.17	0.21	0.18	0.18	0.06	0.22
Starch	**−0**.**38**	**−0**.**67**	**−0**.**48**	0.14	**−0**.**51**	**−0**.**49**
Total carbohydrate	0.00	0.16	0.00	0.16	0.00	0.19
Ratio						
Glucose/fructose	**−0**.**44**	0.23	**−0**.**51**	**−0**.**30**	**−0**.**58**	**−0**.**32**
Sucrose/hexose	0.00	0.00	0.00	0.03	0.00	0.16
Hexose/starch	**0**.**44**	**0**.**63**	**0**.**54**	**0**.**54**	**0**.**62**	**0**.**46**
Sucrose/starch	**0**.**66**	**0**.**56**	**0**.**67**	**0**.**55**	**0**.**58**	**0**.**56**

In the total aboveground harvested material, which comprised the combined stem and leaf, fewer significant differences between genotypes were observed in either 2011 or 2012 at both sites for the concentration of carbohydrates **[see Supporting Information—Table S1]**. The regression analyses of the 2012 data produced some similar results to the analysis of leaf and stem separately **[see Supporting Information—Table S2]**. The ratio of hexose : starch and sucrose : starch produced significant correlations of 0.3–0.5 with canopy height and final yield at both sites and the glucose : fructose ratio produced significantly negative correlations with growth rate and final yield at both sites **[see Supporting Information—Table S2]**.

The similarity in results between the field study at Aberystwyth and the replicated field site at Harpenden strongly supports the case that carbohydrate partitioning between different pools is genetically regulated and the abundance of carbohydrates and particularly the ratios between different pools can be used as indicators of biomass potential.

## Discussion

At Aberystwyth, *M.*× *giganteus* (Gig-311) exhibited a distinct carbohydrate profile showing a high ratio of hexose : starch and sucrose : starch and a low ratio of glucose : fructose owing to a greater abundance of fructose. The same was observed at Harpenden but many of these features were also observed in Sac-5 at this site. This is particularly interesting when the final yields of the different genotypes were compared; whilst Gig-311, Sin-11 and Goliath all yielded similarly (<50 g DW difference) between the two sites, Sac-5 yielded an average of 200 g per plant (30 %) more at Harpenden. Therefore, when Sac-5 was high yielding it possessed a similar carbohydrate fingerprint to Gig-311. This provides strong evidence that a high ratio of sucrose : starch and hexose : starch is an indicator, or biomarker, of high productivity. These finding support our first and second starting questions; that individual carbohydrates and the partitioning between different pools can be correlated with biomass traits.

Although the precise biological parents of *M.*× *giganteus* are unknown, it is known that this hybrid was derived from a cross between a Japanese, diploid *M. sinensis* and a Japanese, tetraploid *M. sacchariflorus* (Linde-Laursen 1993; Hodkinson *et al*. 2002). We therefore considered that the Sin-11 and Sac-5 genotypes used in this study were phylogenetically similar to the parental types of Gig-311. In terms of carbohydrate composition and partitioning between leaf and stem, Gig-311 is more similar to Sac-5 and so it appears that if the carbohydrate profile was to be used to screen new *M. sacchariflorus*× *M. sinensis* hybrids, selecting individuals that have inherited their carbohydrate metabolome from the *M. sacchariflorus* parent rather than *M. sinensis* would be the informed choice. Fewer differences between genotypes were observed in the leaf material, compared with the stem and differences tended to be less consistent between sites, therefore partitioning between the leaf and stem is a poor indicator of yield potential.

The carbohydrate concentrations observed in our study are similar to those reported in a *M.*× *giganteus* genotype grown in July in the Mid-Western USA where starch concentrations in 2-year-old plants of the *M.*× *giganteus,* ‘Illinois’, clone were reported to be 9 mg g^−1^ DW in the leaf and 8 mg g^−1^ DW in the stem (de Souza *et al*. 2013), which is comparable with our findings of <10 mg g^−1^ DW in both organs of 3- and 4-year-old plants. Furthermore, the ratio of total hexose : starch observed in the stem was ∼2 : 1 both in our study and that of the ‘Illinois’ clone when measured in July (de Souza *et al*. 2013).

In a study of the cell wall composition of 244 *Miscanthus* genotypes over 3 years, significant differences between years were observed, but these differences were smaller than genotypic differences between *M. sinensis*, *M. sacchariflorus* and hybrid genotypes (Allison *et al*. 2011). This supports our finding that the non-structural carbohydrate profile is genetically controlled as the cell wall is produced from the partitioning of carbohydrates from the soluble pool into the structural biomass. If structural carbohydrate partitioning is genetically controlled, it is therefore logical that non-structural carbohydrate partitioning would also be. The similarity in the performance of the genotypes between the two sites used in this study and the comparability of our current study with previously published results, e.g. de Souza *et al.* (2013) and Purdy *et al.* (2014), demonstrates that the results are robust; under peak growing conditions, high yielding genotypes have a low starch and high hexose phenotype and a high ratio of soluble sugars to starch.

In a study to investigate the effects of elevated CO_2_, *M.*× *giganteus* was observed to increase in leaf hexoses and decline in starch abundance in both leaf and stem (de Souza *et al*. 2013). In some ways, our results mirror this because Gig-311 had the highest fructose abundance and lowest starch at both sites and Goliath, the genotype with the highest starch, contained the lowest hexose concentration in the leaf and total aboveground (combined leaf and stem). Our data and that of de Souza *et al*. (2013) therefore suggest that a negative relationship exists between hexoses, particularly fructose, and starch. The enzyme pathways that synthesize sucrose and starch are highly similar, the main difference being the spatial separation; sucrose is synthesized in the cytosol and starch in the chloroplast. The synthesis of both requires glucose-1-phosphate as an intermediate substrate and the two processes are competitive, with carbon flow being directed by the concentration of triose phosphate and orthophosphate (P_i_) (Pettersson and Ryde-Pettersson 1989). Owing to the competitive nature of starch and sucrose biosynthesis it is logical to conclude that if hexose phosphates are being driven into starch metabolism then the abundance of sucrose would be lower. This is what we observed in the stem tissues of the different genotypes; in Sac-5 and Gig-311 stems, the proportion of sucrose was greater and starch was lower (as inferred from the sucrose: starch ratios) whereas in Sin-11 and Goliath the opposite was true.

The stem of all genotypes at both sites contained glucose : fructose ratios of >2 : 1, with the exception of the stem of Gig-311, which was closer to 1 : 1. Fructose is exclusively produced by the metabolism of sucrose through the action of both sucrose synthase (SUSY) and INVs (Koch 2004). Glucose, however, is produced both from the metabolism of sucrose through the action of INVs (but not SUSY) and through the metabolism of starch (Koch 2004; Smith *et al*. 2005). Therefore, as fructose is the predominant product from the metabolism of sucrose, a high abundance of glucose relative to fructose is indicative that a high rate of starch turnover is occurring (Häusler *et al*. 1998). This result shows that quantifying glucose and fructose indirectly provides information about the abundance of starch. This is important because whereas soluble sugars can be quantified with relative ease, especially with the use of automated high-performance liquid chromatography or gas chromatography–mass spectrometry systems, starch analysis requires more rigorous pre-treatment of samples before the addition of specific enzymes to digest the starch into glucose (Smith and Zeeman 2006). Therefore, if the ratio of glucose to fructose could be used as a biomarker of productivity in place of starch abundance, this would increase the speed, and lower the cost, of analysing large numbers of samples.

The low concentration of starch in the fastest growing and highest yielding genotypes is in agreement with previous studies in *Arabidopsis* and maize that have also identified a negative relationship between starch and biomass (Rocher 1988; Sulpice *et al*. 2009). In the *Arabidopsis* study, negative relationships were also observed between sucrose and biomass, but the opposite was found in the study on maize (Rocher 1988; Meyer *et al*. 2007). The authors of the *Arabidopsis* study concluded that when (non-structural) carbohydrates are in greater abundance it is due to reduced utilization, i.e. slower growth (Sulpice *et al*. 2009). However, as the study in maize showed a positive correlation with sucrose abundance and growth rate (Rocher 1988) and we have shown that Gig-311 has a high hexose phenotype, it suggests that the correlations between carbohydrate and biomass may differ between monocots and dicots and/or between C3 and C4 species.

A greater number of individual genotypes are required to test whether the observations between carbohydrates and biomass observed in our study hold true in larger populations and/or families of *Miscanthus*. Further studies are now underway to determine whether the carbohydrate phenotypes identified in our study of Gig-311 can be used as biochemical markers of productivity by expanding our research into a larger number of hybrid progeny from *M. sacchariflorus*× *M. sinensis* crosses. If relationships between carbohydrate concentration and biomass traits (e.g. growth rate) are confirmed, it will be important to address at what stage in the plants' development this relationship can be observed. Our study was carried out on plants in their third and fourth complete growing season, but if the relationship could be identified in seedlings, or 1-year-old plants, then such markers could be used as a method of selection to accelerate breeding programmes in a similar way to the potential of molecular markers. However, if relationships between carbohydrates and biomass do not become apparent until maturity, it would be of limited benefit to use them directly as markers but they could prove important quantitative traits for genome mapping studies. This method has been successfully used in *Arabidopsis* where a co-localization of quantitative trait loci for metabolites and dry weight has been discovered in two different mapping families (Calenge *et al*. 2006; Meyer *et al*. 2007). The use of genetical metabolomics has largely been used as a tool in model species such as *Arabidopsis* but notable successes have been achieved in crop species, e.g. the identification of gene polymorphisms that confer greater vitamin A content in maize (Harjes *et al*. 2008). Therefore, the potential of using the glycome (complete carbohydrate profile) as a quantitative trait for either yield improvement or quality improvement (to facilitate fermentation) is also a real possibility.

## Conclusions

Our results show that highly productive genotypes of *Miscanthus* have a distinctive carbohydrate phenotype, showing a high ratio of soluble sugars to starch and a low concentration of starch in the stem. This phenotype was consistent across years and sites, demonstrating that the phenotype is genetically controlled and would be suitable for genetic mapping studies. The abundance of different carbohydrates and particularly the ratios between carbohydrate groups in the stems will be used in a larger screen to test whether these measurements are suitable biochemical markers of productivity in new *Miscanthus* hybrids.

## Sources of Funding

This work was supported by the BBSRC Sustainable Bioenergy Centre (BSBEC) grant (BB/G016216/1) working within the BSBEC-BioMASS (http://www.bsbec-biomass.org.uk/) programme of the centre, the BBSRC Energy Grasses & Biorefining Institute Strategic Programme (BBS/E/W/00003134) at IBERS and the Cropping Carbon Institute Strategic Programme at Rothamsted Research and Ceres Inc.

## Contributions by the Authors

S.J.P. conceived the starting hypotheses for this manuscript. S.J.P., A.L.M., J.C., I.D. and J.C.-B. designed the experiments. A.L.M. carried out the carbohydrate analyses. S.J.P., A.L.M. and J.C. carried out the field work and sample collection. S.J.P. wrote the paper with significant input and editing from all other authors.

## Conflict of Interest Statement

The BSBEC-BioMASS project was primarily funded by the Biotechnology and Biological Sciences Research Council (BBSRC) but also received funding from an industrial partner, Ceres Inc. The funders had no role in study design, data collection and analysis, decision to publish, or preparation of the manuscript.

## Supporting Information

The following additional information is available in the online version of this article –

**Figure S1.** The total monthly rainfall from May–July 2011 and 2012 and the 19-year averages for both sites. The 1981–2010 rainfall data are sourced from the UK Met Office which has weather stations located at both research institutes.

**Table S1.** The concentration of carbohydrates in the total aboveground in July 2011 and July 2012 at Aberystwyth and Harpenden.

**Table S2.** Linear regression analysis of carbohydrate concentrations and ratios against the biomass traits in the total above ground biomass in 2012 at Aberystwyth and Harpenden.

Additional Information
