# Supplementary material for: Non-structural carbohydrate profiles and ratios between soluble sugars and starch serve as indicators of productivity for a bioenergy grass
Source: AoB Plants. 2015 May 12;7:plv032. doi: 10.1093/aobpla/plv032 (PMC5024741; doi:10.1093/aobpla/plv032)
Supplement: Additional Information [file supp_7_plv032_index.html]

Non-structural carbohydrate profiles and ratios between soluble sugars and starch serve as indicators of productivity for a bioenergy grass — Non-structural carbohydrate profiles and ratios between soluble sugars and starch serve as indicators of productivity for a bioenergy grass — Additional Information 

# Non-structural carbohydrate profiles and ratios between soluble sugars and starch serve as indicators of productivity for a bioenergy grass

## Additional Information

Additional Information

**Files in this Data Supplement:**

- Supplementary Figure 1 - tif file
- Supplementary Table 1 - tif file
- Supplementary Table 2 - tif file
