# Supplementary figures and images for: Non-structural carbohydrate profiles and ratios between soluble sugars and starch serve as indicators of productivity for a bioenergy grass
Source: AoB Plants. 2015 May 12;7:plv032. doi: 10.1093/aobpla/plv032 (PMC5024741; doi:10.1093/aobpla/plv032)

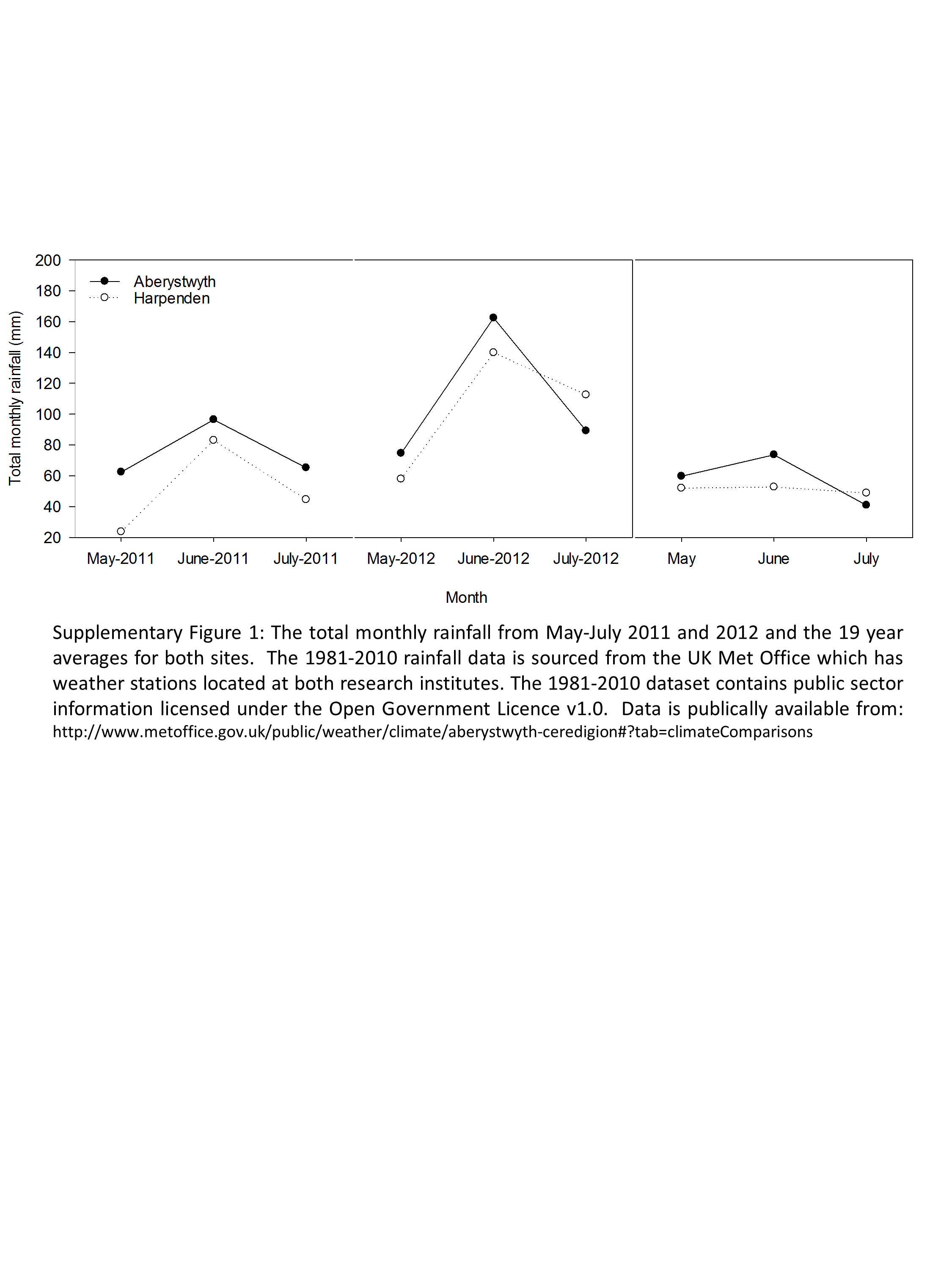

Supplement: Additional Information [file supp_plv032_plv032supp_fig1.tif]

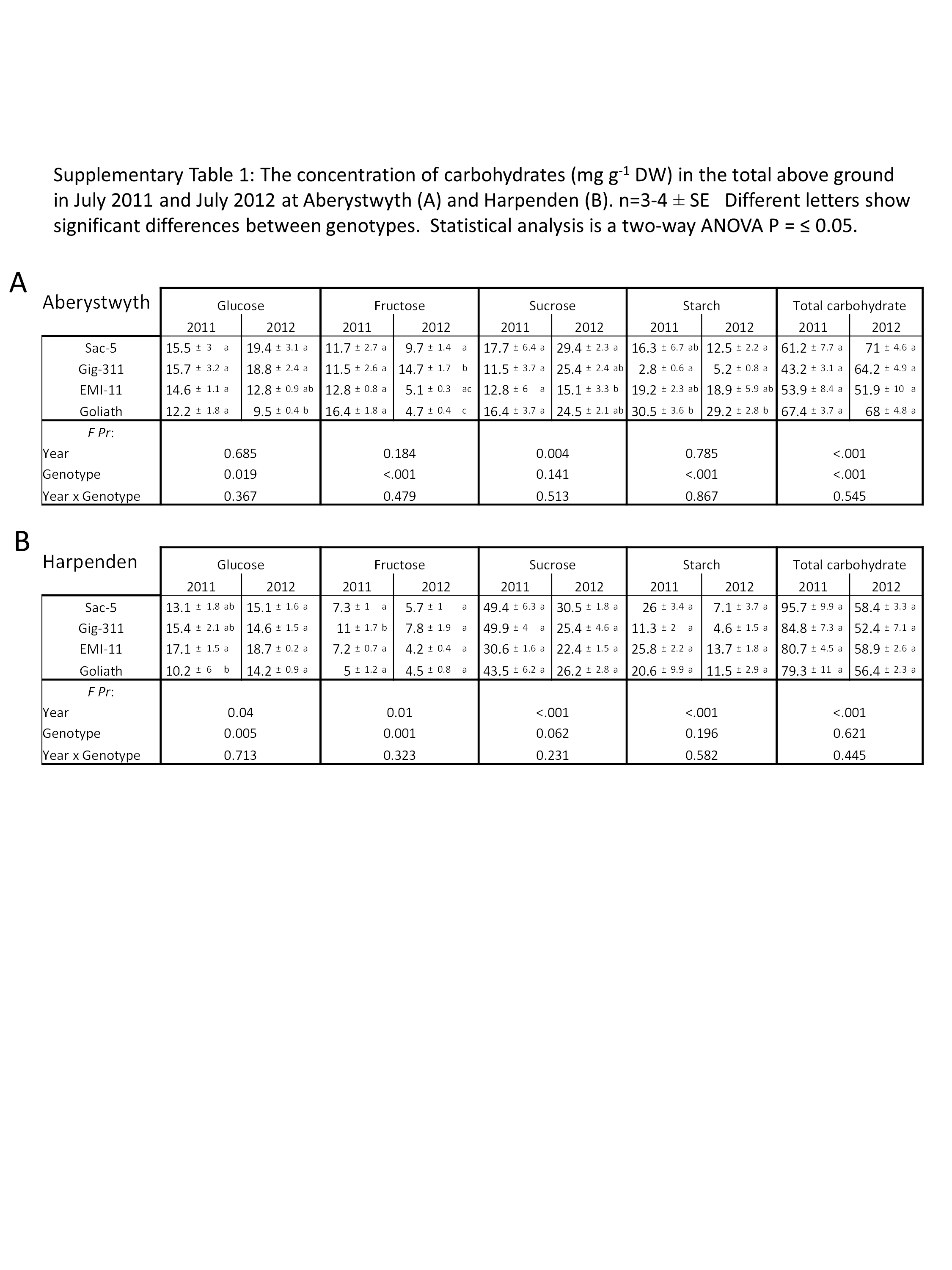

Supplement: Additional Information [file supp_plv032_plv032supp_table1.tif]

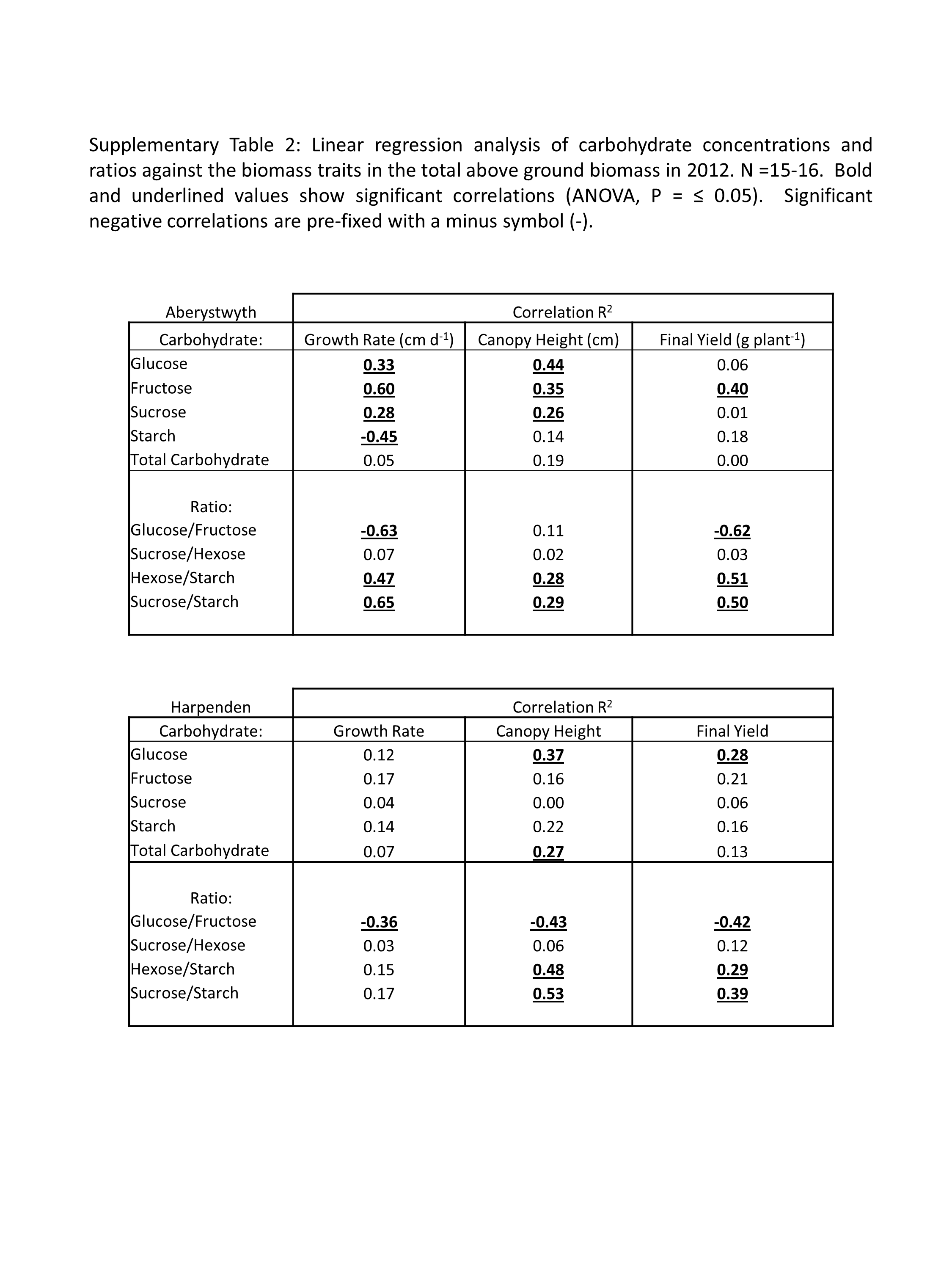

Supplement: Additional Information [file supp_plv032_plv032supp_table2.tif]
